# Exploiting Capture Diversity for Performance Enhancement of ALOHA-Based Multi-Static Backscattering Systems in the 6G Perspective

**DOI:** 10.3390/s21155070

**Published:** 2021-07-27

**Authors:** Roberto Valentini, Piergiuseppe Di Marco, Fortunato Santucci

**Affiliations:** 1 Department of Information Engineering, Computer Science and Mathematics, University of L’Aquila, 67100 L’Aquila, Italy; piergiuseppe.dimarco@univaq.it (P.D.M.); fortunato.santucci@univaq.it (F.S.); 2 Center of Excellence EX-EMERGE, 67100 L’Aquila, Italy

**Keywords:** backscattering communications, random channel access, cross-layer analysis

## Abstract

In this paper, we consider the emerging context of ALOHA-based multi-static backscattering communication systems. By assuming an architecture consisting of a set of passive backscattering nodes, an illuminator, and a set of spatially dislocated receivers, we firstly propose a cross-layer framework for performance analysis. The model jointly accounts for the shared wireless channel, including fading and capture effect, and channel contention strategy, which is regulated by a Framed Slotted ALOHA protocol. Furthermore, based on the inherent macroscopic diversity offered by the multi-static settings, we introduce the concept of capture diversity, which is shown to enable multiple packet detection in slots with multiple transmissions. In order to characterize the multiple access interference and approximate the capture probabilities, we enforce a log-normal approximation of the inverse Signal-to-Interference Ratio that relies on moment matching. Numerical results show the impact of deployment scenarios and the relative positions of illuminator, backscattering nodes, and receivers on the system normalized throughput. We show how the number of detection points impacts the system performance under various channel conditions. Moreover, the accuracy of the proposed approximation rationale is validated via Monte Carlo simulations. Finally, we analyze the optimal frame length in the presence of capture diversity.

## 1. Introduction

Ultra-high energy efficiency (UHEE) is a new paradigm for massively populated and densely composed communication networks at the heart of the massive growth of Internet-of-thing (IoT) applications, and constitutes a complex challenge for beyond 5G (B5G) wireless systems. B5G refers to the network specifications and requirements represented by the era beyond the launch of 5G and before the anticipated launch of 6G [1]. In this context, the need of protocols that require minimum overhead and simplified access mechanism has pushed forward the design of uncoordinated random access solutions [2] based on slotted-ALOHA strategies in combination with the Non-orthogonal Multiple Access (NOMA) paradigm [3,4,5]. Moreover, ultra-high energy efficiency for B5G systems is achieved by considering the design of extremely simple and passive device architectures, and backscattering communication has emerged as one of the most promising technologies [6].

The backscattering communication principle has found wide diffusion in the context of Radio Frequency Identification (RFID) applications, which were originally supported by a mono-static architecture consisting of an active device (i.e., carrier emitter or reader), with physically co-located transmitting and receiving antenna, and passive transponders (i.e., passive devices or tags) that have data to be collected by the reader. The passive nature of the tags (i.e., the absence of a dedicated and durable power source) imposes a very simple design and almost-zero computational capabilities. The reader transmits a continuous wave signal to the tags that in turn scavenge a portion of the incident power to operate an antenna’s load impedance switching modulator [7]. Nevertheless, mono-static systems suffer from very limited coverage due to the inherent inefficiencies of the scavenging circuitry at the device side [8]. To overcome coverage limitations, multi-static architectures have been proposed [9], where a set of networked carrier emitters/readers support communication with backscattering devices either cooperatively or concurrently. Albeit the increased system complexity, multi-static solutions guarantee robustness in terms of both energy provisioning and communication reliability.

Furthermore, the inherent simplicity of passive devices’ architecture poses substantial limitations to the design of the system protocol stack. For instance, the devices’ inability to perform channel sensing restricts the applicability of complex collision resolution mechanisms. At the same time, the inability to handle complex synchronization mechanisms for the devices make orthogonal multiple access protocols, such as Orthogonal Frequency Division Multiple Access (OFDMA), not attractive. In general, promising channel access for passive devices is based on simple random access protocols, such as ALOHA, characterized by very low overhead and high energy efficiency.

With the objective of extending the backscattering communication paradigm to the context of ultra-high energy efficient communication, system design is naturally oriented at multi-static solutions due to the offered extended coverage and flexibility. In addition, the energy required to power up the passive nodes can be harvested from non-dedicated ambient sources [10], thus avoiding the deployment of dedicated power beacons. More importantly, the presence of multiple receivers inherently gives rise to macroscopic diversity that can be effectively exploited to enhance system performance and take advantage of the propagation features that characterize the communication environment. Specifically, the difference in the relative distance among passive devices and backscattering receivers, and the different fading realizations observed at different detection points, can concur at reducing the detrimental effect of interference and allow for simultaneous detection of multiple packets [11,12].

### 1.1. Paper Contributions

In this paper, we propose a cross-layer framework for the performance analysis of ALOHA-based multi-static backscattering communication systems. The considered architecture consists of a single illuminator (i.e., carrier emitter), which provides powering signals for a set of passive backscattering nodes, and a set of spatially deployed receivers. Given the considered scenario, we propose the following contributions:

- We present a framework that describes propagation phenomena and their impact on medium access control protocol dynamics. Specifically, the channel abstraction considers log-normal fading statistics and includes cascade communication links that affect both nodes’ powering and backscattering signals’ detection. To mitigate multiple access interference, we assume that channel contention is driven by a Framed Slotted ALOHA (FSA) [13] protocol and we show how propagation impairments affect detection probability and collision occurrence.
- We characterize the statistical properties of the multiple access interference in terms of Signal-to-Interference Ratio (SIR), which does not admit a closed-form representation in terms of Probability Density Function (PDF) in the proposed settings. To this aim, we approximate the SIR by means of a log-normal random variable for which we determine the unknown parameters via moment matching. The proposed approximation rationale is based on the technique described in [14] and here extended to the peculiar features of the backscattering propagation cascade channel, including nodes’ powering.
- By exploiting the proposed approximation, we are able to study the capture effect at a given receiver in terms of capture distribution. Furthermore, when exploiting the spatial diversity offered by multiple detection points we describe the concept of capture diversity that was first introduced in our prior work [11] in the context of RFID systems. In particular, we show how different samples of fading and interference processes available at different receivers can be exploited in multi-static systems to resolve simultaneous nodes’ transmissions during collision events.
- Based on the proposed framework, we study the performance of the considered multi-static architecture with focus on different parameters such as fading severity, number of deployed receivers and network topology. Moreover, we discuss the impact of FSA settings on the optimal achievable performance in the presence of capture diversity and propagation channel impairments. Specifically, we show how the capability of multi-static systems to resolve simultaneously transmitted data packets substantially reduces the optimal frame length of the FSA and definitely improves channel access performance.

### 1.2. Related Works

Backscattering communications have traditionally found their main application field in RFID systems. In that context, cross-layer frameworks for mono-static ALOHA-based systems have been proposed to analyze and optimize the identification performance metrics, such as identification time. Refs. [15,16,17,18] propose the analysis and optimization of slotted ALOHA channel access schemes in the presence of the capture effect [19]. However, in the aforementioned studies the capture probability is given as a model parameter, thus resulting in a strong abstraction of the underlying channel. Furthermore, power outage, which is a peculiar limitation for passive communication devices, is not taken into account. The capture effect and the effect of fading spatial correlation on mono-static RFID systems have been studied in [20], based on a more realistic propagation channel model. In [21] the first and second order statistics of the fading are derived for both time-invariant and time-variant channels and used to build a Markov chain model of an ALOHA-based RFID system. A theoretical analysis of throughput and bit error rate for multi-tag systems, including propagation channel statistics, has been performed in [22]. Capture effect has also been included in recent studies dealing with cross-layer performance analysis of ALOHA-type one-way communications. For instance, in [23], the combined effect of interference cancellation, packet segmentation, slot slicing and capture is analyzed by means of a theoretical framework that allows to derive capture probability in both fast and slow Rayleigh fading conditions.

To overcome inherent coverage limitations of mono-static backscattering systems, multi-static architectures have been proposed, where a set of networked illumitators/readers operate either cooperatively or concurrently to identify a population of passive tags. In [24,25] the authors deal with the derivation of a stable readers scheduling algorithm that is designed for dynamic environments, where the number of arriving tags in the illumination area changes over time. In [26] a heuristic multi-channel scheduling algorithm for coordination of multiple readers is derived with the advantage of being adaptable to existing multiple access schemes for single reader scenarios. In [27], an optimization-based distributed scheme is proposed in order to avoid unpractical centralized coordination mechanisms among readers. In [28], an approach based on readers’ cooperation is proposed to handle collisions. The recent work in [29] proposes a novel reader scheduling policy that substantially outperforms state of the art solutions. Finally, a novel Code Division Multiple Access (CDMA) collaborative protocol for multi-reader RFID systems is proposed in [30], where the CDMA paradigm is exploited to combat reader-to-reader interference. Nevertheless, all the aforementioned studies focus on the derivation of scheduling policies to resolve interference among readers and do consider neither physical layer peculiarities nor cross-layer interactions. Furthermore, the reception diversity opportunity offered by the multi-static setting is not considered. The recent study in [9] proposes a framework for multi-static backscattering systems that includes accurate models for the physical layer and the propagation channel, and is not limited to RFID applications. Transmit diversity is considered in order to extend the system coverage. A comprehensive analysis is conducted, which includes the derivation of Bit Error Rate (BER) performance bounds, information and energy outage probabilities. The theoretical analysis is then corroborated by experimental demonstration of overall performance improvements of multi-static systems over the mono-static counterpart. In [31] a multi-static RFID architecture is proposed, where MAC layer operations are distributed over a set of networked Software Defined Radio (SDR) illuminators.

ALOHA-based protocols that exploit capture effect and interference cancellation have also been proposed in the context of uncoordinated NOMA cellular systems [32]. In [33], Coded Slotted Aloha (CSA) performance is analyzed in the presence of capture effect, whereas [34] considers an energy-constrained NOMA scheme based on power and packet diversity and implementing interference cancellation. Motivated by the renewed visibility of backscattering communications in the context of beyond 5G and massive IoT arenas, many recent studies explore the possibility of considering NOMA solution in the backscattering communication frame and extend the applicability of traditional RFID paradigms. In [35], the authors proposed the adoption of the power-domain NOMA (i.e., multiplexing the backscattering nodes in different regions or with different backscattered power levels) to enhance the spectrum efficiency of the backscattering communication system. For an ALOHA-type random access, the work in [36] introduced a framework for backscattering-based intelligent sensing with machine learning. In [37], the authors derive a closed-form BER expressions for a bi-static backscattering communication system employing NOMA with imperfect successive interference cancellation under a Nakagami-m fading channel.

Finally, multi-antenna solutions, that are widely used in traditional one-way wireless channels, have been considered to improve performance and increase capacity of backscattering communication systems. Recent studies in this direction propose Multiple Input Multiple Output (MIMO) architectures considering multiple antennas at either node side or illuminator/receiver side. Specifically, traditional space-time coding (STC), beamforming-like techniques, receive and transmit diversity for the forward link, have been adapted for backscattering MIMO systems [38,39,40,41,42,43].

The rest of this paper is organized as follows. In Section 2 we introduce the system model and describe the considered multi-static architecture, the propagation channel abstraction and the channel access contention protocol. In Section 3 we propose a methodology to characterize the statistics of multiple access interference via moment matching approximation. Moreover, we introduce and describe the concept of capture diversity. In Section 4 we provide numerical results, analyze the performance of the considered multi-static system and validate the proposed cross-layer approach. Finally, Section 5 concludes the paper.

### 1.3. Notation

Random variables are denoted by sans-serif-style upper-case letters (e.g., X). Sets are denoted by calligraphic upper-case letters (e.g., X) unless otherwise stated. The operators Pr(·) and E[·] represent probability measure and expectation, respectively.

## 2. System Model

### 2.1. System Architecture

**Figure 1:**
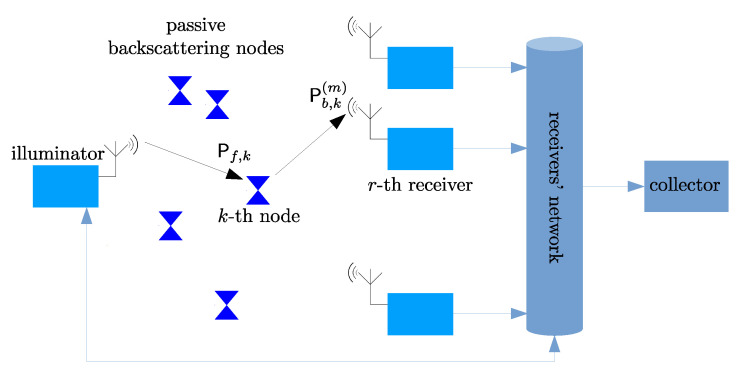
System architecture.

We consider the system architecture depicted in Figure 1, consisting of a set of *K* backscattering passive nodes, an illuminator, and a set of *M* receivers. The illuminator triggers the passive nodes by sending a *beacon* signal that instructs the nodes to acquire and communicate data (e.g., sensory data, identification codes, etc.). The illuminator transmit power is fixed and we denote it by $P_{\mathrm{tx}}$. The data acquired at the *k*-th tag is communicated by modulating the reflected signal by a simple impedance switching logic. Given their passive nature, the nodes extract the power required to activate the switching modulator from the incident signal and this can only occur if the incident power at the *k*-th node’s antenna, which we denote as $P_{f,k}$, is sufficiently high. The modulated backscattered signals are detected by the receivers at different spatial locations. The power reflected by the *k*-the node and received at the *m*-th receiver’s antenna is denoted as $P_{b,k}^{\left(m\right)}$. Communication occurs over a shared channel affected by fading. As a consequence, both the received power at the backscattering nodes and the received power at the backscattering receivers undergo random fluctuations that affect the communication quality. Moreover, access to the shared medium is coordinated by the illuminator that implements a FSA protocol to mitigate multiple access interference. The remainder of the proposed analysis is based on the following assumptions:

*A*_1_ information about the successful detection of a node’s data is available immediately at the illuminator. This can be achieved by a high speed network between the receivers and the illuminator. Nonetheless, the characterization of this high speed network is beyond the scope of this paper and is not explicitly included here;
*A*_2_ communication occurs over a slow varying channel, where fading is time invariant within a sufficiently long time interval;
*A*_3_ fading components are log-normally distributed. Although it may appear limiting, this assumption is well suited to describe different propagation phenomena, such as shadow fading and, in several indoor scenarios, even multipath fading [44].
*A*_4_ fading components do not exhibit spatial correlation;
*A*_5_ the network operates in saturation conditions, where each node has always data to send.
*A*_6_ buffering and/or re-transmission policies are not included in our framework, so as data packets are dropped if not successfully delivered in a slot.

The propagation channel abstraction and the medium access scheme model, including the effect of the channel impairments on the dynamic of the multiple access coordination are described in the reminder of this section.

### 2.2. Propagation Channel Abstraction

Different from traditional one-way channels, backscattering channels exhibit composite fading effects, given that the received signal at a generic receiver is affected by the product of two cascade fading components that describe the forward link (i.e., illuminator-node link) and the backscattering link (i.e., node-receiver link), respectively.

Under the settings described in assumptions *A*_2_ and *A*_3_, the power received by the *k*-th node through the forward link is given as

(1) $$\begin{array}{c} P_{f,k}=P_{\mathrm{tx}}\alpha_{k}e^{S_{f,k}}, \end{array}$$

with k=1,…,K, where $\alpha_{k}=c_{0}/d_{f,k}^{k_{0}}$, $d_{f,k}$ is the distance between the illuminator and the *k*-th tag, $c_{0}$ is the frequency-dependent path-loss at reference distance of 1 m, $k_{0}$ is the path-loss exponent, and $S_{f,k}∼N\left(0,\sigma^{2}\right)$ is zero-mean Gaussian random variable that describes fading on the forward link.

The level of the received power at the node determines whether or not the node is able to modulate the reflected signal. Specifically, we consider a simplified node abstraction, where the node is able to reflect all the incident power (in practice, due to loss phenomena of the harvesting circuitry, only a fraction of the incident power can be backscattered by the node. Moreover, the portion of the reflected power is, in general, a non-linear function of the incident power itself [45]. We remark that a more refined node abstraction can be safely included in our framework, without substantial modifications.) if $P_{f,k}$ is larger than the node’s sensitivity, which we denote as $\gamma_{h}$. On the contrary, when the received power is below the sensitivity threshold, the scattered power is equal to zero. Therefore, the received power at the *m*-th receiver can be expressed as

(2) $$\begin{array}{c} P_{b,k}^{\left(m\right)}=\left\{\begin{array}{cc} \beta_{m,k}P_{f,k}e^{S_{b,k}^{\left(m\right)}} & P_{f,k}>\gamma_{h}\\ 0 & \mathrm{otherwise}, \end{array}\right. \end{array}$$

with m=1,…,M. Here, $\beta_{m,k}=c_{0}/d_{b,k}^{k_{0}}$ where $d_{b,k}$ is the distance between the *k*-th node and the *m*-th receiver and $S_{b,k}^{\left(m\right)}∼N\left(0,\sigma^{2}\right)$ describes the fading in the backscattering link. By substituting Equation (1) in Equation (2), we get

(3) $$\begin{array}{c} P_{b,k}^{\left(m\right)}=\left\{\begin{array}{cc} P_{\mathrm{tx}}D_{r,k}e^{Y_{k}^{\left(m\right)}} & P_{f,k}>\gamma_{h}\\ 0 & \mathrm{otherwise}, \end{array}\right. \end{array}$$

where $D_{m,k}=\alpha_{k}\beta_{m,k}$ and $Y_{k}^{\left(m\right)}=S_{f,k}+S_{b,k}^{\left(m\right)}$. Given the physical separation between the illuminator’s antenna and the generic receiver’s antenna, which in our settings is much higher than the operative wavelength, the fading components $S_{f,k}$ and $S_{b,k}^{\left(m\right)}$ can be considered uncorrelated [46] and, thus independent. Therefore, $Y_{k}^{\left(m\right)}$ is still a zero-mean Gaussian random variable, with variance $2\sigma^{2}$. In addition, according to assumption *A*_4_, the random variables $S_{f,k}$ and $S_{b,k}^{\left(r\right)}$, for k=1,…,N and m=1,…,M, are independent.

### 2.3. Random Channel Access Model

**Figure 2:**
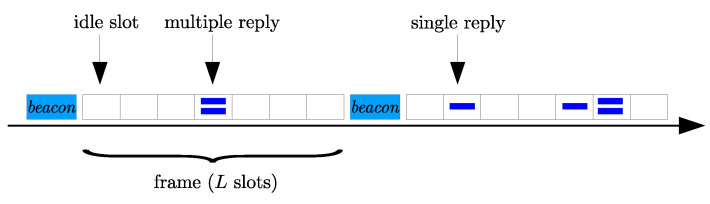
Framed Slotted ALOHA.

Communication occurs over a shared channel where multiple access is regulated by a FSA protocol. Referring to assumption *A*_5_, nodes always have backlogged data that must be delivered. To start the communication session, the illuminator triggers the beginning of a frame, which has a duration of *L* time slots, by broadcasting a *beacon* signal. The *beacon* is also used to communicate the frame length to the backscattering nodes. Upon receiving the beacon, the backscattering nodes randomly select a slot within the frame and transmit data in the allotted slot. This process may results in idle slots, where no node transmits, single node transmissions or multiple transmissions, where interference may cause loss of information. The dynamic of the medium access strategy is illustrated in Figure 2 and formally described in what follows.

In order for the *k*-th node to be able to transmit data in a frame, it must correctly receive the *beacon*. This occurs if the received power $P_{f,k}$ is above the node’s sensitivity threshold $\gamma_{h}$. Therefore, due to the fading affecting the forward links, only a subset A of nodes in the network can effectively communicate in a given frame. Denoting by $P_{A}$ the event where the nodes in A successfully receive the *beacon* signal, that is $P_{A}=\left\{P_{f,k}>\gamma_{h},\forall k\in A\right\}$, the probability that the set of nodes A is able to communicate in a frame can be expressed as

(4) $$\begin{array}{cc} \mathrm{Pr}(P_{A}) & =\prod_{i\in A}\mathrm{Pr}\left(P_{f,i}>\gamma_{h}\right)\prod_{j\in K\backslash A}\left[1-\mathrm{Pr}\left(P_{f,j}>\gamma_{h}\right)\right]\\ \; & =\prod_{i\in A}\mathrm{Pr}\left(S_{f,i}>\ln\frac{\gamma_{h}}{\alpha_{i}P_{\mathrm{tx}}}\right)\prod_{j\in K\backslash A}\left[1-\mathrm{Pr}\left(S_{f,j}>\ln\frac{\gamma_{h}}{\alpha_{j}P_{\mathrm{tx}}}\right)\right]\\ \; & =\prod_{i\in A}\left[1-F_{S}\left({\hat{\gamma }}_{h,i}\right)\right]\prod_{j\in K\backslash A}F_{S}\left({\hat{\gamma }}_{h,j}\right). \end{array}$$

where K is the set of backscattering nodes, ${\hat{\gamma }}_{h,i}=\ln\frac{\gamma_{h}}{\alpha_{i}P_{\mathrm{tx}}}$ and $F_{S}\left(x\right)$ is the Cumulative Distribution Function (CDF) of a zero-mean Gaussian random variable with variance $\sigma^{2}$. Due to power outage, the nodes in K\A are not able to communicate until a new beacon is issued by the illuminator.

Within a frame, the nodes in A independently initializes a slot counter, whose value is sampled at random from the interval [0,L−1] and decremented in each slot. Nodes associated with a null counter gain access to the channel and start transmitting. Denoting by I⊂A the set of transmitting nodes in a slot, the following mutually exclusive outcomes can be experimented on the receivers’ side:

1. Idle Slot: |I|=0, that corresponds to the case where no node has a null slot counter, and thus no transmission occurs;
2. Single transmission slot: |I|=1, that corresponds to the event where only one node has a slot counter equal to zero. In this case, the received signal is correctly detected by the *m*-th receiver if the backscattered power $P_{b,k}^{\left(m\right)}$ is larger than the receiver sensitivity threshold, which we denote as $\gamma_{d}$. Then, conditioned to the event $P_{A}$, the probability for the single transmitted packet to be successfully detected can be expressed as
  (5) $$\begin{array}{cc} \mathrm{Pr}(P_{b,k}^{\left(m\right)}>\gamma_{d}|P_{A}) & =\mathrm{Pr}(P_{b,k}^{\left(m\right)}>\gamma_{d}|P_{f,k}>\gamma_{h})\\ \; & =\mathrm{Pr}(P_{b,k}^{\left(m\right)}>\gamma_{d}|P_{f,k}>\gamma_{h})\\ \; & =\frac{\mathrm{Pr}(P_{b,k}^{\left(m\right)}>\gamma_{d},P_{f,k}>\gamma_{h})}{\mathrm{Pr}(P_{f,k}>\gamma_{h})}\\ \; & =\frac{\mathrm{Pr}(P_{b,k}^{\left(m\right)}>\gamma_{d},S_{f,k}>{\hat{\gamma }}_{h,k})}{F_{S}\left({\hat{\gamma }}_{h,k}\right)} \end{array}$$
  where we exploited the independence among fading components. Then, it may happen that the power at the *m*-th receiver input is below the receiver sensitivity threshold, thus yielding a data packet loss.
3. Multiple transmission slot: |I|>1, that corresponds to the case where multiple nodes gain access to the channel in the same slot. In this case, the received backscattered signal at the generic receiver consists of the superimposition of multiple interfering transmissions. This event is typically interpreted as a collision, nevertheless, due to the so-called *capture effect*, a node’s data packet can be successfully detected at the *m*-th receiver despite the interference. A successful packet detection can occur when the backscattered power from a node is sufficiently large if compared to the power of the interfering signals. More precisely, this occurs when the SIR measured at a given receiver for a given node’s packet is larger than the so-called SIR threshold (or *capture ratio*), which we denote as $\gamma_{\mathrm{SIR}}$. Formally, by defining the SIR for the *k*-th tag at the *m*-th receiver as
  (6) $$\begin{array}{c} {\mathrm{SIR}}_{k}^{\left(m\right)}=\frac{P_{b,k}^{\left(m\right)}}{\sum_{j\in I}^{j\neq k}P_{b,j}^{\left(m\right)}}, \end{array}$$
  the *k*-th nodes’s packet is successfully detected at the *m*-th receiver with probability
  (7) $$\begin{array}{c} p_{c,k}^{\left(m\right)}=\mathrm{Pr}\left({\mathrm{SIR}}_{k}^{\left(m\right)}>\gamma_{\mathrm{SIR}}|P_{A}\right), \end{array}$$
  then, the probability of having a successful packet detection at the *m*-th receiver is given as
  (8) $$\begin{array}{c} p_{\mathrm{succ}}^{\left(m\right)}=\sum_{k\in I}p_{c,k}^{\left(m\right)}. \end{array}$$
  A collision occurs when the SIR associated to all the replying nodes is below the SIR threshold, and the collision probability at the *m*-th receiver can be trivially determined as
  (9) $$\begin{array}{c} p_{\mathrm{coll}}^{\left(m\right)}=1-p_{\mathrm{succ}}^{\left(m\right)}. \end{array}$$
  According to assumption *A*_6_, if a collision occurs, the data packets associated to all interfering nodes are dropped with consequent loss of information. The characterization of the multiple access interference and the derivation of the probability in Equation (7) are deferred to Section 3.

To measure the communication performance of the considered system we define the Frame Success Rate (FSR) as the number of successfully delivered packet normalized by the frame length *L*. More precisely, conditioned on a specific set of active nodes in a frame, the FSR can be expressed as

(10) $$\begin{array}{c} FSR|P_{A}=\frac{n_{\mathrm{succ}}|P_{A}}{L}, \end{array}$$

where $n_{\mathrm{succ}}$ is the number of delivered packets. Then, the expected FSR, $R_{s}$ can be obtained as

(11) $$\begin{array}{c} R_{s}=\sum_{A\subset K}E\left[FSR|P_{A}\right]\mathrm{Pr}\left(P_{A}\right). \end{array}$$

Observe that $R_{s}$ is affected by the selected frame length *L*. Assuming that the number of nodes in the network is known at the illuminator, the frame length can be set at the optimal theoretical value L=K. Nevertheless, propagation channel effects induce a shift on the optimal value of the frame length. Indeed, due to node activation failures in a specific frame, the actual number of nodes contending the channel is lower than *K*, and thus a frame set to *K* slots would increase the number of idle slots. Similarly, capture effect also impacts on the optimal frame duration. Specifically, multiple transmission slots does not necessarily result in collisions, and thus the frame length could be set to a value lower than *K* to achieve optimal performance. Albeit a formal derivation of the optimal performance is beyond the scope of our analysis, we include a discussion on optimal frame length in Section 4.

## 3. Multiple Access Interference Characterization and Capture Diversity

In order to study the FSR for the considered multi-static system, a crucial step is the statistical characterization of the interference that allows to determine the capture probability defined in Equation (7). To this aim, we consider a multiple transmission slot with a generic set of transmitting nodes I, with |I|>1. Conditioned on the event $P_{A}$, the capture probability at the *m*-th receiver for the *k*-th node is

(12) $$\begin{array}{cc} p_{c,k}^{\left(m\right)} & =\mathrm{Pr}\left(\frac{P_{b,k}^{\left(m\right)}}{\sum_{j\in I}^{j\neq k}P_{b,j}^{\left(m\right)}}>\gamma_{\mathrm{SIR}}|P_{A}\right)\\ \; & =\frac{\mathrm{Pr}\left(\sum_{j\in I}^{j\neq k}\frac{P_{b,j}^{\left(m\right)}}{P_{b,k}^{\left(m\right)}}<\frac{1}{\gamma_{\mathrm{SIR}}},P_{f,i}>\gamma_{h},\forall i\in A\right)}{\mathrm{Pr}\left(P_{f,i}>\gamma_{h},\forall i\in A\right)}\\ \; & =\frac{\mathrm{Pr}\left(\sum_{j\in I}^{j\neq k}\frac{P_{b,j}^{\left(m\right)}}{P_{b,k}^{\left(m\right)}}<\frac{1}{\gamma_{\mathrm{SIR}}},P_{f,i}>\gamma_{h},\forall i\in I\right)}{\mathrm{Pr}\left(P_{f,i}>\gamma_{h},\forall i\in I\right)}\\ \; & =\frac{\mathrm{Pr}\left(\sum_{j\in I}^{j\neq k}\frac{D_{m,j}}{D_{m,k}}e^{Y_{j}^{\left(m\right)}-Y_{k}^{\left(m\right)}}<\frac{1}{\gamma_{\mathrm{SIR}}},S_{f,i}>{\hat{\gamma }}_{h,i},\forall i\in I\right)}{\prod_{i\in I}\left[1-F_{S}\left({\hat{\gamma }}_{h,i}\right)\right]}. \end{array}$$

The derivation of the above probability requires the statistics of the random variable

(13) $$\begin{array}{c} X_{k}^{\left(m\right)}=\sum_{j\in I}^{j\neq k}\frac{D_{m,j}}{D_{m,k}}e^{Y_{j}^{\left(m\right)}-Y_{k}^{\left(m\right)}}, \end{array}$$

with k∈I, which represents the inverse of the SIR. However, the random variable $X_{k}^{\left(m\right)}$ is given as a weighted sum of log-normal random variables, which has an unknown distribution (i.e., the PDF of $X_{k}^{\left(m\right)}$ does not exist in closed form). Therefore, we approximate the inverse SIR with a log-normal random variable as

(14) $$\begin{array}{c} X_{k}^{\left(m\right)}\approx e^{T_{k}^{\left(m\right)}}, \end{array}$$

with $T_{k}^{\left(m\right)}∼N\left(\eta_{T_{k}^{\left(m\right)}},\sigma_{T_{k}^{\left(m\right)}}^{2}\right)$, where $\eta_{T_{k}^{\left(m\right)}}$ and $\sigma_{T_{k}^{\left(m\right)}}^{2}$ are unknown parameters that can be determined via moment matching. Specifically, we first compute the first and second order moment of $X_{k}^{\left(m\right)}$ as

(15) $$\begin{array}{cc} \eta_{X_{k}^{\left(m\right)}} & ≜E[X_{k}^{\left(m\right)}]\\ \; & =E\left[\sum_{j\in I}^{j\neq k}\frac{D_{m,j}}{D_{m,k}}e^{Y_{j}^{\left(m\right)}-Y_{k}^{\left(m\right)}}\right]\\ \; & =\sum_{j\in I}^{j\neq k}\frac{D_{m,j}}{D_{m,k}}E\left[e^{Y_{j}^{\left(m\right)}-Y_{k}^{\left(m\right)}}\right]\\ \; & =\frac{e^{2\sigma^{2}}}{D_{m,k}}\sum_{j\in I}D_{m,j}. \end{array}$$

(16) $$\begin{array}{cc} \delta_{X_{k}^{\left(m\right)}} & ≜E\left[{X_{k}^{\left(m\right)}}^{2}\right]=E\left[\sum_{j\in I}^{j\neq k}\frac{D_{m,j}}{D_{m,k}}e^{Y_{j}^{\left(m\right)}-Y_{k}^{\left(m\right)}}\sum_{l\in I}^{l\neq k}\frac{D_{m,l}}{D_{m,k}}e^{Y_{l}^{\left(m\right)}-Y_{k}^{\left(m\right)}}\right]\\ \; & =\frac{1}{D_{m,k}^{2}}\sum_{j\in I}\sum_{l\in I}^{l\neq j}D_{m,j}D_{m,l}E\left[e^{Y_{j}^{\left(m\right)}-Y_{k}^{\left(m\right)}+Y_{l}^{\left(m\right)}-Y_{k}^{\left(m\right)}}\right]\\ \; & =\frac{e^{8\sigma^{2}}}{D_{m,k}^{2}}\sum_{j\in I}D_{m,j}^{2}+\frac{e^{6\sigma^{2}}}{D_{m,k}^{2}}\sum_{j\in I}^{k\neq j}\sum_{l\in I}^{l\neq k,j}D_{m,j}D_{m,l}, \end{array}$$

then, recalling that the first and second order moment of the log-normal random variable $e^{T_{k}^{\left(m\right)}}$ can be expressed as

(17) $$\begin{array}{c} m_{1}=e^{\eta_{T_{k}^{\left(m\right)}}+\frac{1}{2}\sigma_{T_{k}^{\left(m\right)}}^{2}}, \end{array}$$

and

(18) $$\begin{array}{c} m_{2}=e^{2\eta_{T_{k}^{\left(m\right)}}+2\sigma_{T_{k}^{\left(m\right)}}^{2}}, \end{array}$$

respectively, we impose

(19) $$\begin{array}{c} \left\{\begin{array}{c} \eta_{X_{k}^{\left(m\right)}}=m_{1}\\ \delta_{X_{k}^{\left(m\right)}}=m_{2}, \end{array}\right. \end{array}$$

which gives

(20) $$\begin{array}{c} \eta_{T_{k}^{\left(m\right)}}=\ln\frac{\eta_{X_{k}^{\left(m\right)}}^{2}}{\sqrt{\delta_{X_{k}^{\left(m\right)}}}}, \end{array}$$

and

(21) $$\begin{array}{c} \sigma_{T_{k}^{\left(m\right)}}^{2}=\ln\frac{\delta_{X_{k}^{\left(m\right)}}}{\eta_{X_{k}^{\left(m\right)}}^{2}}. \end{array}$$

Once $\eta_{T_{k}^{\left(m\right)}}$ and $\sigma_{T_{k}^{\left(m\right)}}^{2}$ are known, the conditional probability in Equation (12) can be approximated as

(22) $$\begin{array}{c} p_{c,k}^{\left(m\right)}\approx \frac{\mathrm{Pr}\left(T_{k}^{\left(m\right)}<\hat{\gamma },S_{f,i}>{\hat{\gamma }}_{h,i},\forall i\in I\right)}{\prod_{i\in I}\left[1-F_{S}\left({\hat{\gamma }}_{h,i}\right)\right]}, \end{array}$$

where we defined $\hat{\gamma }=-\ln\gamma_{\mathrm{SIR}}$. To compute the approximated capture probability in Equation (22) we first need to determine the covariance between $T_{k}^{\left(m\right)}$ and $S_{f,i}$ for all i∈I, which we define as

(23) $$\begin{array}{c} c_{T_{k}^{\left(m\right)},i}=Cov\left(T_{k}^{\left(r\right)},S_{f,i}\right). \end{array}$$

The conditional probability in Equation (12) can be expressed by deriving the correlations

(24) $$\begin{array}{cc} \delta_{X_{k}^{\left(m\right)},i} & ≜E[X_{k}^{\left(m\right)}e^{S_{f,i}}]\\ \; & =E\left[\sum_{j\in I}^{j\neq k}\frac{D_{m,j}}{D_{m,k}}e^{Y_{j}^{\left(m\right)}-Y_{k}^{\left(m\right)}}e^{S_{f,i}}\right]\\ \; & =\frac{1}{D_{m,k}}\sum_{j\in I}^{j\neq k}D_{m,j}E\left[e^{Y_{j}^{\left(m\right)}-Y_{k}^{\left(m\right)}+S_{f,i}}\right]\\ \; & =\left\{\begin{array}{cc} \frac{e^{\frac{3}{2}\sigma^{2}}}{D_{m,k}}\sum_{j\in I}^{j\neq k}D_{m,j} & k=i\\ \frac{e^{\frac{5}{2}\sigma^{2}}}{D_{m,k}}\sum_{j\in I}^{j\neq k,j\neq i}D_{m,j}+\frac{D_{m,i}}{D_{m,k}}e^{\frac{7}{2}\sigma^{2}} & k\neq i. \end{array}\right. \end{array}$$

Being

(25) $$\begin{array}{c} E\left[e^{T_{k}^{\left(m\right)}}e^{S_{f,i}}\right]=E\left[e^{T_{k}^{\left(m\right)}}\right]E\left[e^{S_{f,i}}\right]e^{c_{T_{k}^{\left(m\right)},i}}, \end{array}$$

with i∈I, the covariance between $T_{k}^{\left(m\right)}$ and the fading component on the *i*-th forward link is thus obtained as

(26) $$\begin{array}{c} c_{T_{k}^{\left(m\right)},i}=\ln\frac{\delta_{X_{k}^{\left(m\right)},i}}{e^{\eta_{T_{k}^{\left(m\right)}}+\frac{1}{2}\left(\sigma_{T_{k}^{\left(m\right)}}^{2}+\sigma^{2}\right)}}. \end{array}$$

The approximated capture probability in Equation (22) can be expressed in terms of multi-variate Gaussian CDF and can be efficiently evaluated by means of standard numeric integration approaches [47]. The proposed approximation is an extension of the generalized method proposed in [14], which is derived from the Fenton-Wilkinson approach. It has been shown that Fenton-Wilkinson method accuracy is low for σ>4 dB [48] and, for such values of σ, the log-normal PDF poorly fits the distribution of the linear combination of log-normal random variables. Nevertheless, the tails distribution of the sum is still well approximated even for larger values of σ [49]. Consequently, the proposed method is expected to exhibit good accuracy when applied to the considered problem, since we are interested in computing the capture probability that is expressed in terms of tail distribution. This sapect is validated in Section 4, where results obtained enforcing the proposed approximation method are compared with a Monte Carlo simulation, which converges to the actual distribution of the weighted sum of log-normal random variables. Finally, we want to remark that the described method is well suited to approximate even randomly weighted sums of log-normal random variables [14]. Then, the proposed rationale can be used to accurately approximate capture probability in scenarios characterized by more general product channels, which include different fading statistics (e.g., Exponential distribution for Rayleigh fading, Non-central Chi-square distribution for Rice fading, Gamma distribution for Nakagami fading, etc.). More precisely, regardless of the fading statistics considered for the single two-way channel, the SIR is approximated by a log-normal random variable with proper parameters. The inclusion of additional random components in Equation (2) would clearly affect the first and second order moment of $X_{k}^{\left(m\right)}$ and, in turn, the parameters of the approximating log-normal random variable. Nevertheless, this effect can be easily accounted in our framework by varying σ.

### Capture Diversity

For any multiple transmission slot, it is possible to associate a capture distribution to each receiver. Formally, given a generic receiver *m* and the interferes set I, the capture distribution is given by the capture probabilities $p_{c,k}^{\left(m\right)}$, with k∈I, including the collision probability $p_{\mathrm{coll}}^{\left(m\right)}$. Since backscattered signals are received at different spatial locations, and thus experiment different attenuation and fading realizations, the SIR associated to a generic node *k* differs at different receivers. This implies that the capture probabilities $p_{c,k}^{\left(m\right)}$ distribute differently from one receiver to another. Therefore, data packet from different nodes can be captured simultaneously at different receivers, yielding multiple packet reception during multiple transmission slots. We refer to this as capture diversity.

**Figure 3:**
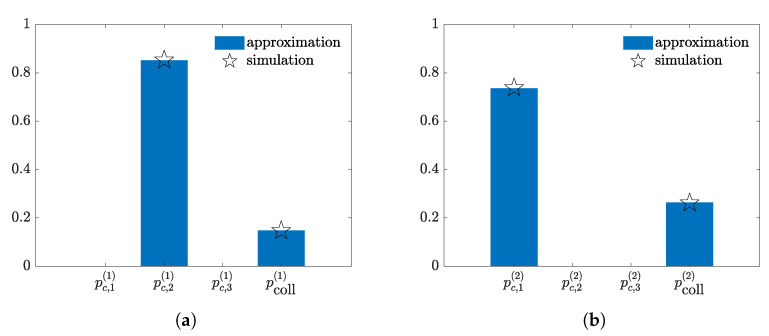
Example of capture distributions for σ=2 dB in a scenario with K=50 nodes, M=2 receivers and 3 randomly selected transmitting nodes in a slot. (**a**) Receiver 1. (**b**) Receiver 2.

To illustrate capture diversity, we consider an example where a multiple transmission slot occurs in presence of two spatially dislocated receivers. We assume a set of 50 backscattering nodes disseminated at random within a circular area of radius 5 m around the illuminator. The receivers are placed at antipodal positions with respect to the illuminator, at the border of the circular area. We assume that three randomly selected nodes transmit simultaneously in a slot and we compute the approximated capture distributions relying on Equation (22). The capture distributions associated to the two receivers for σ=2 dB are depicted in Figure 3a,b, respectively. To show the accuracy of the proposed approximation we also derive the capture distribution by means of Monte Carlo simulations, which converge to the actual value of the capture probabilities. Observe that the second node’s packet has a good chance of being captured by the first receiver, whereas, the first node’s packet is likely captured at the second receiver. Thus, the probability of successfully receiving two data packets simultaneously in the same slot is substantially high. Note also that the accuracy of the approximation method is very good for the selected value of σ.

**Figure 4:**
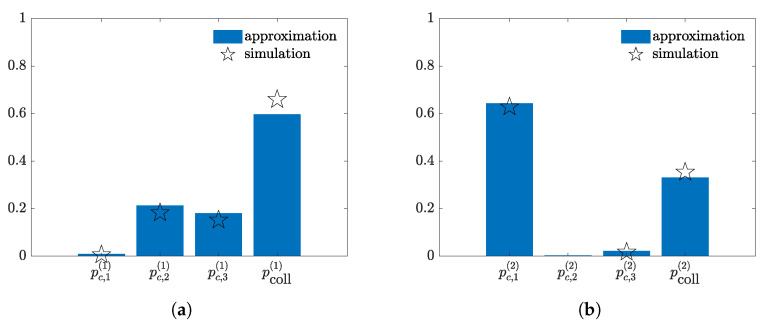
Example of capture distributions for σ=6 dB in a scenario with K=50 nodes, M=2 receivers and 3 randomly selected transmitting nodes in a slot. (**a**) Receiver 1. (**b**) Receiver 2.

A second example is considered, where the scenario is kept fixed and σ is set to 6 dB. The approximated and simulated capture distributions associated to the first and second receiver are reported in Figure 4a,b, respectively. In this case, the higher fading spread reflects on the spread of the capture distributions. Indeed, the first receiver have now a non zero probability of capturing either the second node’s packet or the third node’s packet. The second receiver, instead, has a good chance of capturing the first node’s data packet. Note that the larger value of σ also affects the accuracy of the proposed approximation method. However, although a lower accuracy is associated to higher value of the fading spread, the approximation still perform well.

In order to quantify the impact of capture diversity, we define the Capture Rate (CR) as the probability of having capture events in multiple transmission slots. Formally, conditioned on a specific set of active nodes in a frame, the CR can be expressed as

(27) $$\begin{array}{c} CR|P_{A}=\frac{\sum_{i=1}^{L}\phi_{i}|P_{A}}{n_{\mathrm{mr}}}, \end{array}$$

where $\phi_{i}$ is a binary flag that is high if capture occurs in a slot and $n_{\mathrm{mr}}$ is the number of multiple transmission slots in a frame. Then, the expected capture rate can be obtained as

(28) $$\begin{array}{c} C=\sum_{A\subset K}E\left[CR|P_{A}\right]\mathrm{Pr}\left(P_{A}\right). \end{array}$$

We remark that capture diversity differs from conventional micro-diversity concept, where redundancy is exploited by means of signal combining approaches that aim at maximizing detection performance. Instead, capture diversity is given by the combination of spatial macro-diversity diversity and the dynamic of the medium access protocol. Specifically, the randomness induced by the channel access negotiation scheme is such that simultaneously transmitting nodes are typically placed at different distances from the receivers, thus contributing to the near-far phenomenon, which encourage capture effect. Therefore, capture diversity can be interpreted as a form of network-level diversity.

## 4. Results

In order to analyze the performance of the considered multi-static backscattering communication system and to validate the proposed approximation method we consider the expected FSR and the expected CR as defined in Equations (11) and (28), respectively. Specifically, we study the complex and often counter-intuitive impact of relevant system parameters, such as the number of disseminated receivers *M*, the network topology, and the fading spread σ on the FSR and the CR. Moreover, we also analyze the impact of the frame length *L* on the optimal system performance.

We considered a reference network scenario where backscattering nodes are disseminated within a circle of radius $d_{I}$, and the illuminator is placed at the center of the circle, which we refer to as the illumination area. Unless otherwise stated, we assume that the *N* = 50 nodes are located at random positions within the circle, and the FSR is obtained by averaging over 100 different realizations of nodes spatial dissemination. The receivers are placed at fixed positions uniformly dislocated on a circle of radius $d_{IR}$ in the illumination area.

The illuminator transmit power $P_{\mathrm{tx}}$, the nodes sensitivity threshold $\gamma_{h}$, the receivers’ sensitivity threshold $\gamma_{d}$, the SIR threshold $\gamma_{\mathrm{SIR}}$, the operative frequency *f* and the path-loss exponent $k_{0}$ are fixed and the relative values are summarized in Table 1. We observe that the values considered for the aforementioned parameters are compliant with realistic passive backscatterig systems. Specifically, the illuminator transmit power and the operative frequency are set to standard values for passive RFID systems [50]. Similarly, the nodes’ sensitivity threshold is set to a reference value of commercial passive tags and the receivers’ sensitivity threshold is compliant with commercial RFID readers. We further assume that all receivers and all backscattering nodes have the same characteristics, thus $\gamma_{d}$ is the same for all receivers and $\gamma_{h}$ is the same for all the nodes. Finally, the SIR threshold is set to a value commonly assumed in several capture effect models [19]. Although the considered parameters are representative of state-of-the-art backscattering systems, we remark that the proposed framework easily allows to consider different parameters’ sets, which are not addressed in this study.

To obtain the FSR and the CR we enforce the approximation approach described in Section 3. Unless otherwise stated, the frame length *L* is set equal to the number of nodes *K*, which corresponds to the theoretical optimal frame length of FSA in absence of propagation phenomena. Furthermore, to validate the accuracy of the proposed method, we compare the obtained results with Monte Carlo simulation. Specifically, the simulated FSR and CR are obtained by averaging over 1000 Monte Carlo trials.

**Figure 5:**
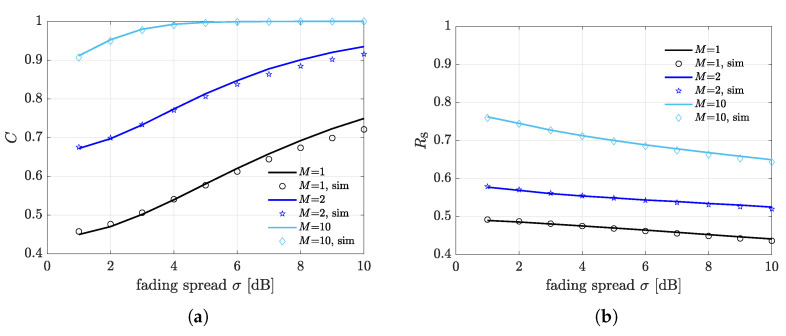
Impact of fading spread σ for different number of deployed receivers. The considered scenario consists of K=50 backscattering nodes and the receivers are located at the border of the illumination area at a distance of $d_{IR}=10$ m from the illuminator. (**a**) Capture rate. (**b**) Frame success rate.

The impact of fading spread σ is illustrated in Figure 5 for different numbers+ of receivers, specifically M=1,2,10. The illumination area radius is set to $d_{I}=10$ m and the receivers are placed at the border of the illumination area (i.e., $d_{IR}=10$ m). The expected capture rate is depicted in Figure 5a. Firstly, we notice that higher fading spread values are beneficial in terms of capture rate. This is due to the fact that a larger fading spread σ induces a larger spread in the capture distribution, which increases chance of capture. Secondly, a higher number of deployed receivers substantially increases the capture rate, as expected. Observe that when *M* is set to 10, the capture rate approaches 1 as σ increases, meaning that any multiple transmission yields at least a correctly detected data packet. The expected FSR is shown in Figure 5b. Interestingly, despite the capture rate increases with σ, the FSR exhibits a diminishing trend with σ. This can be explained by considering nodes powering failure. Specifically, a higher fading spread on the forward link affects the nodes powering probability, which is dominant compared to the increment of capture rate. Consequently, the FRS is generally affected negatively by the fading spread. As expected, increasing the number of deployed receivers substantially increases the FSR due to a higher diversity gain.

Observe that the accuracy of the proposed approximation approach is very high for all the considered values of σ. This confirms that the log-normal approximation can be safely applied for the derivation of capture probabilities. Indeed, although a low accuracy is expected for σ>4, this only occurs if the target is the whole PDF of the weighed sum of log-normal components. Nevertheless, capture probabilities are defined in terms of the tail distribution of the sum, thus, the proposed approach maintains a good accuracy within a wide range of σ values.

**Figure 6:**
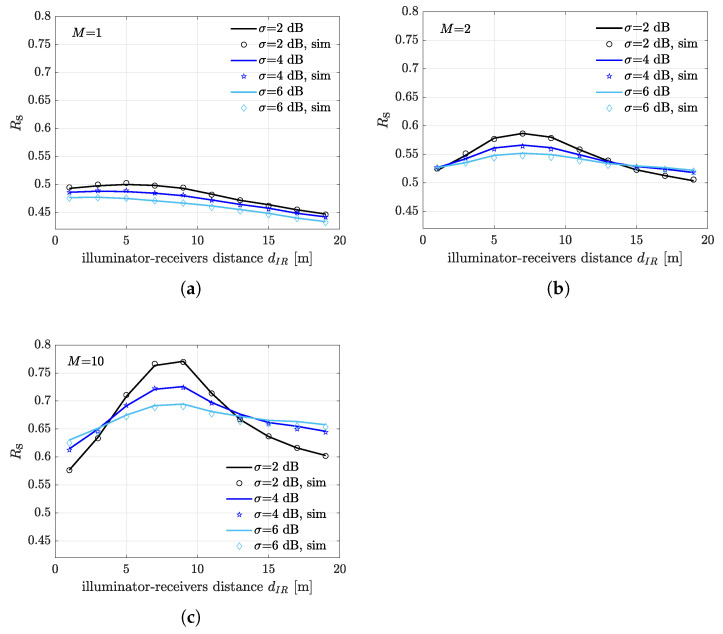
Expected frame success rate as a function of illuminator-receivers distance $d_{IR}$ for different values of σ. (**a**) M=1. (**b**) M=2. (**c**) M=10.

It is expected that the illuminator-receivers distance $d_{\mathrm{IR}}$ has a substantial impact on the system performance. Indeed, the distance between the receivers and the center of the illumination area affects the SIR at the generic receiver and, consequently the capture occurrence. Moreover, $d_{\mathrm{IR}}$ also affect the single reply detection probability defined in Equation (5). The FSR as a function of $d_{\mathrm{IR}}$ is illustrated in in Figure 6, for different values of the fading spread σ and considering different number of deployed receivers. Figure 6a shows the expected FSR for a single deployed receiver. It can be seen that best performance is obtained when the receiver is located within the illumination area and close to the illuminator, especially for σ=6 dB. As $d_{\mathrm{IR}}$ increases the expected FSR diminishes since the spatial configuration is less favorable for capture diversity and the miss-detection probability increases. The case with two antipodal receivers is analyzed in Figure 6b, where the FSR is clearly higher with respect to the case with M=1 due to the increased capture diversity gain. The FSR curve also exhibits an evident maximum when the receivers are placed at a distance that is approximately half of the illumination area radius. We remark that the presence of the maximum is mainly due to the spatial symmetry of the considered network geometry. Specifically, given the fixed dimension of the illumination area, when the receivers are either too close or too far from the illuminator, the nodes distribution is unfavorable to the capture effect. Interestingly, as in the previous case, performance diminishes as the illuminator-reader distance increases. Nevertheless, at a given distance, larger fading spread yields better performance since the higher capture chance better compensates performance loss (i.e., packets miss-detection). Note also that the distance that returns the maximum performance does not depend on the fading severity. A similar trend is observed when M=10 in Figure 6c, where a substantial increment of the FSR is observed due to the additional gain in diversity. Moreover, the effect of the spatial symmetry is exacerbated in this scenario. Indeed, we clearly observe two intersection points that dictate an inversion trend of the FSR with respect to σ. Again, this effect can be explained by first observing that the illumination area is of fixed size, and thus placing receivers either too close or too far from the illuminator results in lower FSR because of bad capture configurations and higher packets miss-detection. On the other hand, capture effect becomes dominating at the intersection points for higher values of the fading spread, thus yielding an increasing FSR as σ increases.

**Figure 7:**
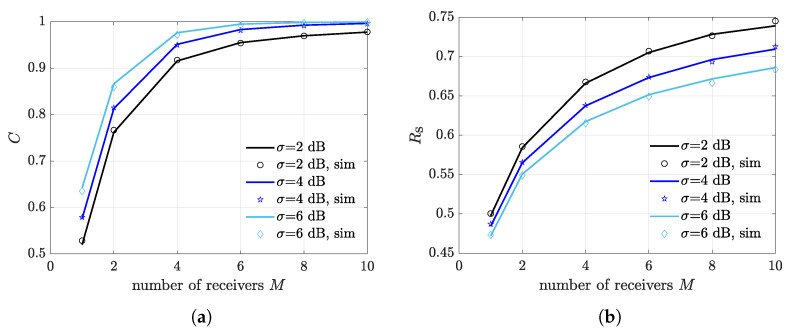
Impact of number of deployed receivers *M*. The considered scenario consists of randomly disseminated nodes and receivers located inside the illumination area. (**a**) Expected capture rate. (**b**) Expected frame success rate.

To analyze how the number of deployed receivers contributes to the diversity gain, we derive the expected CR and the expected FSR as a function of *M*, for different values of the fading spread and a fixed illuminator-receivers distance of $d_{\mathrm{IR}}=6$ m, which has been shown to provide close-to optimum performance in the considered settings. It can be seen from Figure 7a that CR rapidly reaches its maximum as *M* increases. Note that, when σ=2 dB, CR does not saturate to 1, meaning that increasing the number of receivers to much does not guarantee capture occurrence in all multiple transmission slots. As expected, the increasing trend with *M* is also visible for the FSR in Figure 7b. Observe that the saturation effect is less pronounced when referring to the FSR, suggesting that by increasing the number of deployed receivers, the average FSR eventually approaches to 1. This is due to the fact that, during multiple transmission slots, multiple packets reception can occur thanks to capture diversity, therefore a higher *M* produces a higher average number of simultaneously detected packets. Nevertheless, the average number of simultaneously detected packets is also affected by the network topology and, more precisely, by the spatial configuration of the nodes. Indeed, capture diversity depends on the combination of scenario geometry and channel contention mechanism, which randomly selects a subset of transmitting nodes in a slot.

**Figure 8:**
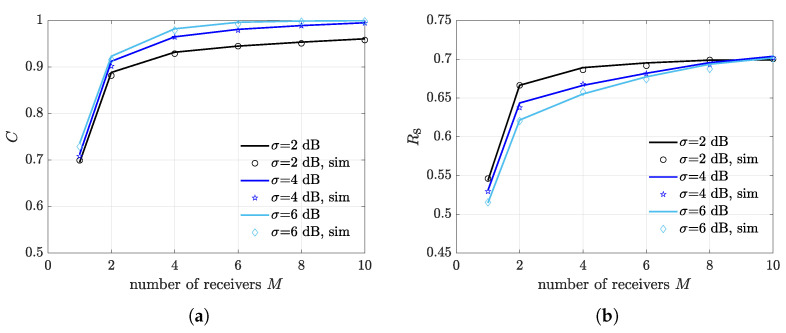
Impact of number of deployed receivers *M*. The considered scenario consists of nodes uniformly distributed over a horizontal line at the center of the illumination area and receivers located inside the illumination area. (**a**) Expected capture rate. (**b**) Expected frame success rate.

To better highlight this aspect, we present the CR and the FSR for a different nodes spatial dissemination in Figure 8a,b, respectively. Specifically, nodes are uniformly distributed on a horizontal line at the center of the illumination area. It can be seen that, in this scenario, the FSR exhibits an evident saturation, especially for σ=2 dB. In this situation, there is no benefit in increasing *M* to much, and satisfactory performance are already obtained for M=2.

**Figure 9:**
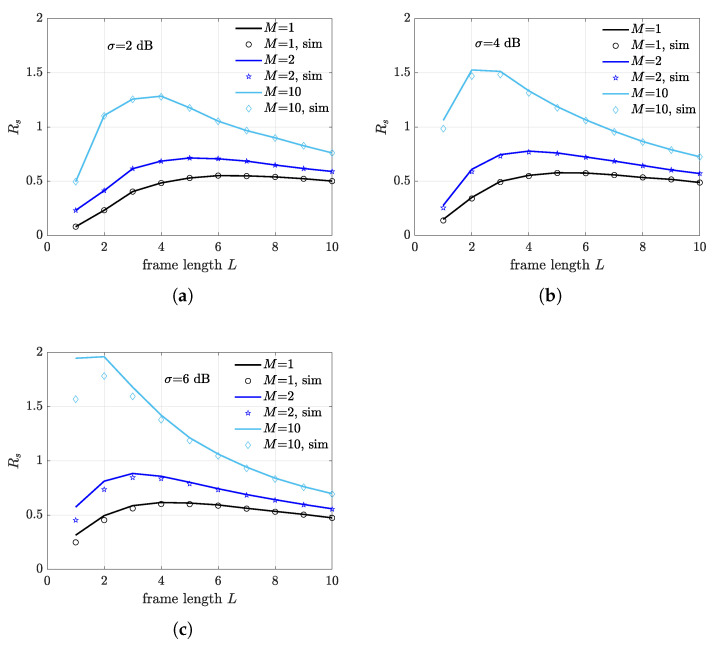
Frame success rate as a function of frame length *L* for different number of deployed receivers. Receivers are placed at the border of the illumination area at a distance of $d_{IR}=10$ m from the illuminator. (**a**) σ=2 dB. (**b**) σ=4 dB. (**c**) σ=6 dB.

All the results derived above assume that the frame length is set to L=K, which corresponds to the theoretical optimal frame length. Nevertheless, due to capture effect, multiple transmissions does not necessarily result in collisions, and, in the considered multi-static setting, multiple packets detection may occur due to capture diversity. Therefore, the actual optimal frame length differs from the theoretical optimum and is shorter than *K*. Observe also that fading and network topology affect the capture probability and, more generally, the capture distribution at different receivers. Consequently, the optimal frame length depends on different system parameters, non-trivially. To unveil the impact of such dependence, we consider a network scenario with K=10 backscattering nodes, where receivers are located at the border of the illumination area at a distance of $d_{IR}=10$ m. Figure 9 shows the expected FSR as a function of *L* for different values of *M* and σ=2 dB (Figure 9a), σ=4 dB (Figure 9b), and σ=6 dB (Figure 9c). As expected, as the number of receivers increases, the frame length associated to the maximum FSR decreases for all the considered values of σ. Note that the fading spread has a twofold impact on the optimal frame length. On the one hand, a larger σ supports a higher diversity gain, and thus a higher probability of multiple packets detection. As a consequence, the optimal frame length decreases as the fading spread increases. On the other hand, a higher σ results in a lower powering probability, thus the reduction of the frame length also results from the reduced average number of powered nodes in a frame.

**Figure 10:**
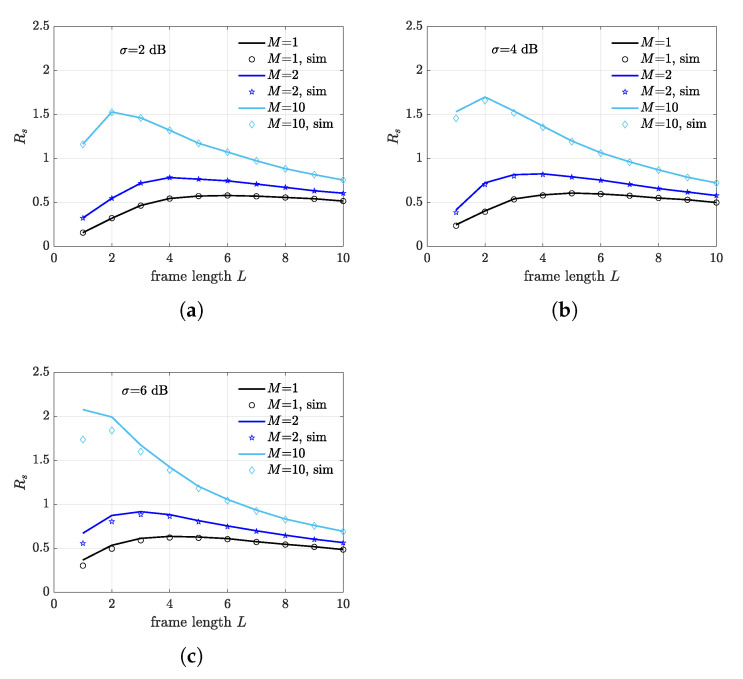
Frame success rate as a function of frame length *L* for different number of deployed receivers. Receivers are placed inside the illumination area at a distance of $d_{IR}=6$ m from the illuminator. (**a**) σ=2 dB. (**b**) σ=4 dB. (**c**) σ=6 dB.

In order to analyze the impact of the illuminator-distance on the optimal frame length, a scenario with receivers located within the illumination area at a distance of $d_{IR}=6$ m is considered in Figure 10. Along with a higher FSR, which is expected at the considered $d_{IR}$, we observe that in this case the optimal frame length is less sensible to fading spread variations. Interestingly, when M=10, which corresponds to the case where the number of receivers is equal to the number of nodes, only two slots are required to achieve optimal performance. The theoretical limit of the expected FSR in the considered scenario is achieved when L=1 and each receiver captures a different tag.

**Figure 11:**
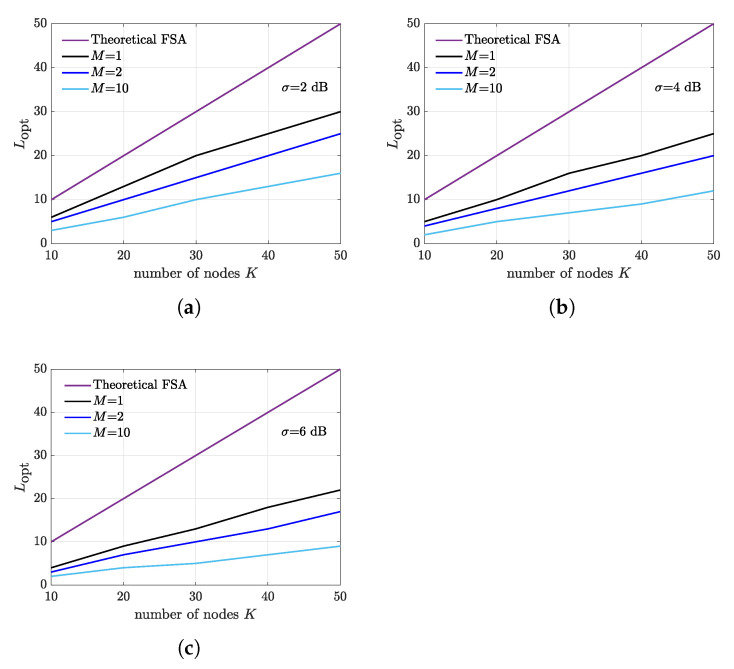
Optimal frame length as a function of *K* for different number of deployed receivers. Receivers are placed at the border of the illumination area at a distance of $d_{IR}=10$ m from the illuminator. (**a**) σ=2 dB. (**b**) σ=4 dB. (**c**) σ=6 dB. Theoretical FSA optimal frame length is also reported for comparison.

Finally, we analyze the optimal frame length as a function of the number of backscattering nodes for M=1,2,10 and σ=2 dB (Figure 11a), σ=4 dB (Figure 11b), and σ=6 dB (Figure 11c). The theoretical optimal frame length value of FSA is also reported in order to highlight the reduction of frame duration in presence of capture effect and capture diversity. As expected, the optimal frame length decreases as the number of receivers increases. Furthermore, the fading spread also has substantial impact on the optimal frame duration.

## 5. Conclusions

In this paper we presented a cross-layer framework for performance analysis of ALOHA-based multi-static backscattering communication systems. We proposed an approximation method based on moment matching in order to characterize multiple access interference and, in turn, derive capture probabilities. By exploiting the receive diversity offered by the set of spatially dislocated receivers, we introduced the concept of capture diversity, consisting in different distribution of capture probabilities at different receivers. We showed that capture diversity enables multiple packets detection during slots with simultaneous transmissions and, consequently, yields substantial performance improvement in terms of normalized throughput. Based on the proposed framework we conducted a comprehensive performance analysis exploring the impact of several system parameters such as, network topology, fading severity and number of deployed receivers. We finally examined the impact of cross-layer interactions on the optimal frame length of the considered FSA protocol. We believe that the proposed analysis lays the foundations for accurate modeling and design of proper protocol and architectural solutions in the field of massive IoT and B5G networks as well.

## Figures and Tables

**Table 1:** Main simulation parameters.

Parameter	Value	Unit
$P_{\mathrm{tx}}$	33	dBm
$\gamma_{h}$	−20	dBm
$\gamma_{d}$	−80	dBm
*f*	915	Mhz
$k_{0}$	2	-
$\gamma_{\mathrm{SIR}}$	6	dB
